# Fatigue and quality-of-life in the year following SARS-Cov2 infection

**DOI:** 10.1186/s12879-022-07517-w

**Published:** 2022-06-13

**Authors:** Peter-Joe Noujaim, Damien Jolly, Claire Coutureau, Lukshe Kanagaratnam

**Affiliations:** 1 CHU Reims, Unité d’Aide Méthodologique, 51100 Reims, France; 2 Université de Reims Champagne-Ardenne, VieFra, 51100 Reims, France; 3 Department of Research and Public Health, Robert Debré Hospital, Reims University Hospitals, Rue du Général Koenig, 51092 Reims, France

**Keywords:** COVID-19, Fatigue, QOL, SF12, Dyspneoa

## Abstract

**Background:**

The SARS-COV2 pandemic has been ongoing worldwide since at least 2 years. In severe cases, this infection triggers acute respiratory distress syndrome and quasi-systemic damage with a wide range of symptoms. Long-term physical and psychological consequences of this infection are therefore naturally present among these patients. The aim of this study was to describe the state of health of these patients at 6 (M6) and 12 months (M12) after infection onset, and compare quality-of-life (QOL) and fatigue at these time-points.

**Methods:**

A prospective cohort study was set up at Reims University Hospital. Patients were clinically assessed at M6 and M12. Three scores were calculated to describe patient’s status: the modified Medical Research Council score (mMRC) used to determine dyspnoea state, the Fatigue Severity Scale (FSS) and the Short Form 12 (SF12) that was carried out to determine the QOL both mentally and physically (MCS12 and PCS12). Descriptive analysis and comparison of scores between M6 and M12 were made.

**Results:**

120 patients completed both follow-up consultations. Overall, about 40% of the patients presented dyspnoea symptoms. The median mMRC score was 1 Interquartile ranges (IQR) = [0–2] at the two assessment. Concerning FSS scores, 35% and 44% of patients experienced fatigue at both follow-ups. The two scores of SF12 were lower than the general population standard scores. The mean PCS12 score was 42.85 (95% confidence interval (95% CI [41.05–44.65])) and mean MCS12 score of 46.70 (95% CI [45.34–48.06]) at 6 months. At 12 months, the mean PCS12 score was 42.18 (95% confidence interval (95% CI [40.46–43.89])) and mean MCS12 score of 47.13 (95% CI [45.98–48.28]). No difference was found between SF12 scores at 6 and 12 months.

**Conclusions:**

This study pinpoints the persistence of fatigue and a low mental and physical QOL compared to population norms even after 1 year following infection. It also supports the claims of mental or psychological alterations due to infection by this new virus, hence a lower overall QOL in patients.

**Supplementary Information:**

The online version contains supplementary material available at 10.1186/s12879-022-07517-w.

## Background

The “CoronaVirus Disease 2019” pandemic (COVID-19) has been rife across the world for almost 2 years now, with over two hundred million people infected and over four million deaths [1].

Health repercussions were rapidly observed, with a saturation of health systems across almost all countries, and an obvious shortage of beds in healthcare units, which left no other choice but new management for emergencies and critical care provision [2], and finally the instatement of emergency staff reinforcements throughout the world, including France with its “plans blancs” [3].

Infection by Severe Acute Respiratory Syndrome—Coronavirus 2 (SARS-CoV2), the COVID-19 agent appears after a median period of 4 days of incubation, mainly with fever and a cough. In severe cases, this infection triggers acute respiratory distress syndrome (ARDS) and quasi-systemic damage with a wide range of symptoms.

Long-term physical and psychological consequences of this infection are therefore naturally present among patients discharged from hospital following initial hospital stays, often including time in a resuscitation unit. These consequences, which remain largely unknown given the novelty of this pandemic, have been monitored and described in several studies, over a 6-month period post-infection at the most. The main physical symptoms observed have been fatigue or muscular weakness, coupled with respiratory disorders, difficulties sleeping, anxiety, and even depression [4].

Fatigue is a symptom that persists after coronavirus infection, as is the case with certain viral infections, and it forms—with the other afore-mentioned symptoms—the post-viral syndrome [5]. This fatigue quite logically affects the patients’ quality-of-life.

According to the World Health Organisation (WHO), quality-of-life is defined as individuals’ perceptions of their position in life in the context of the culture and value systems in which they live, and in relation to their goals, expectations, standards and concerns. This concept has gained considerable importance over the last 30 years, enabling an improvement in patients’ medical, social and economic care. Some studies have shown that following infection by SARS-CoV2, quality-of-life at 12 weeks and at 6 months was diminished among the patients concerned, as well as among their care providers (family members, friends, etc.) [6, 7].

To our knowledge, only two studies have described the state of health of Covid-19 patients beyond 6 months post-infection; one study focused on global symptoms [8] and the other only analysed the patients’ pulmonary state [9], but neither of them carried out analyses on feelings of fatigue or on quality-of-life at that time.

The objective of our study was therefore to describe the state of health (physical symptoms, including fatigue, and psychological symptoms) of patients who had been hospitalised for SARS-CoV2 infection at 6 and 12 months after onset, with a comparative analysis of quality-of-life and fatigue at these time-points.

## Methods

### Study design

A prospective cohort study was set up at Reims University Hospital.

### Population

The inclusion criteria were: patients hospitalised for SARS-CoV2 infection during the health crisis between February 25th 2020 and April 30th 2020, who were aged 18 and over, having French national healthcare cover, and agreeing to participate in the study.

The Covid-19 diagnosis was established by a doctor if the patient tested positive with a PCR test, or on the basis of imagery or a clinical history pointing to this condition.

### Data collection

For all patients, socio-demographic data and medical history were collected at inclusion. Clinical, biological and therapeutic data during hospitalisation was also collected, as well as data relating to the evolution of the disease, such as mortality, resuscitation, institutionalisation or re-admission to hospital.

Data was collected up to 12 months after the initial hospitalisation.

Follow-up consultations at 6 months and 12 months were carried out by the clinicians who had initially provided care to the patients in the Reims university hospital Covid-19 units.

Data concerning clinical examinations, symptoms experienced by patients (dyspnoea, palpitations, asthenia, etc.) and treatments (anticoagulants, psychotropic drugs, etc.) was collected during the follow-up consultations at 6 and 12 months (M6 and M12).

Description and analyses concerned only the 120 patients who attended the two follow-up consultations.

An evaluation of dyspnoea and related experiences among patients was graded at M6 and M12, using the modified Medical Research Council score (mMRC) [10]. This score is generally used for patients with a probable pulmonary condition. It is scored on 5 levels from 0 “no dyspnoea except in the case of sustained effort” to 4 “dyspnoea preventing the patient from leaving the house or occurring when dressing and undressing”.

Fatigue was assessed at M6 and M12 using the Fatigue Severity Scale (FSS) [11]. This scale is used to assess fatigue experienced by patients in their daily lives. It is composed of 9 items, each rated on an ordinal scale, from 1 “does not correspond to me at all” to 7 “corresponds to me perfectly". An average score was calculated (by summing the different item scores and dividing by the number of items) with a global population standard of 2.3, a standard deviation of 0.7 [12] and a threshold at 4 (with a score > 4 indicating great fatigue), as suggested by some authors [13–15]. To our knowledge, this score has been used in three other studies to evaluate the degree of subjective fatigue among patients with infectious diseases (Puumala virus, HCV or Covid 19) [16–18].

Quality-of-life was assessed using the Short Form 12 scale (SF-12) [19] at M6 and M12. This scale is an assessment carried out by patients themselves on their quality of life. It is a shorter version of the SF-36. The different items in this scale are grouped under two subscores:

The PCS 12 score: Physical Component Summary score, grouping items concerning patients’ physical state.

The MCS 12: Mental Component Summary score, grouping items concerning patients’ mental state.

A comparison with values observed in the general population was conducted using an abacus relating to a “normal” state in terms of Quality-of-Life in the French population [19].

### Regulations and ethics

This study constituted research involving humans according to French legislation regulating research on human beings (Jardé legislation).

Approval from the French Research Ethics Board was granted. (No. 3838-RM). The study was registered on the database https://clinicaltrials.gov/ct2/home under No. NCT04553575.

All patients who agreed to take part in the study received full information and signed informed consent.

### Statistical analysis

Qualitative variables were described as numbers and percentages, and quantitative variables were described via means or standard deviations, or as medians and interquartile ranges, depending on the variable distribution.

For comparisons of the scales and the scores between M6 and M12, Wilcoxon’s and McNemar’s tests for paired series were used. A p value < 0.05 was considered significant.

All analyses were conducted using R studio® Version 4.0.5 and SAS Version 9.4 (Institute Inc., Cary, NC, USA).

## Results

Formal diagnoses of SARS-CoV2 infection were made for 479 patients, who were included in the cohort.

**Fig. 1:**
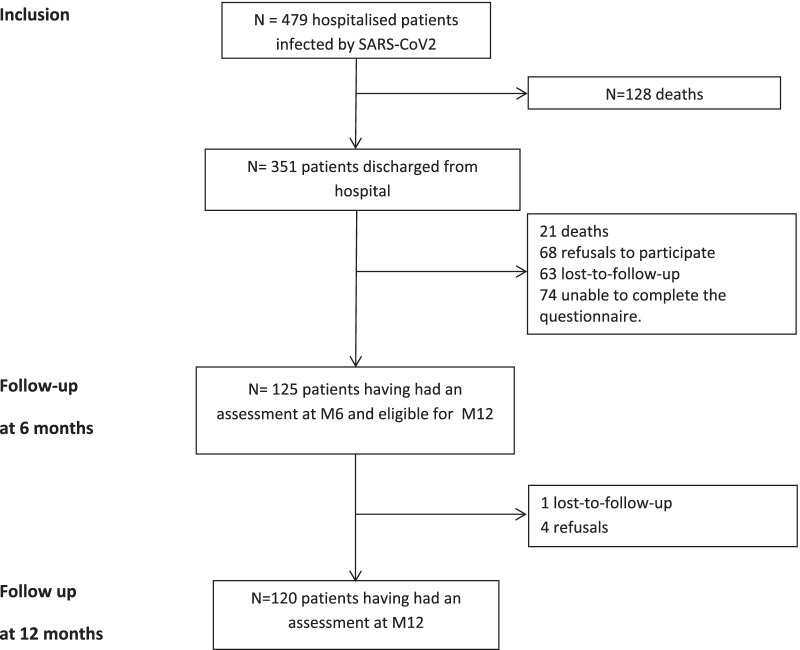
Flow chart for patient follow-up and their attendance for assessment at M6 and M12

After hospitalisation, 128 deaths had occurred. Between hospitalisation discharge and the 6-month assessment 17 deaths had occurred, and there were 16 refusals or individuals lost to follow-up for a total of 318 eligible patients, of whom 193 were excluded (Fig. 1). In all, 120 patients had a follow-up assessment at 6 months and 120 at 12 months.

The initial characteristics of the patients who took part in both follow-up assessments are described in Table 1.

**Table 1:** Initial characteristics of the disease during hospitalisation of patients assessed at M6 and M12 (n = 120)

Variables	Number (%)	Median [IQR]
Socio-demographic characteristics		
Age		63.50 [54.00–71.25]
> 70 years old	32 (26.67)	
Men	65 (54.17)	
Live at home	120 (100.00)	
Symptoms		
Fever	92 (76.67)	
Cough	88 (73.33)	
Dyspnoea	80 (66.67)	
Headache	19 (15.83)	
Diarrhoea	46 (38.30)	
Anorexia	20 (16.67)	
Ageusia	12 (10.00)	
Anosmia	17 (14.17)	
Muscle pains	34 (28.33)	
Abdominal pains	9 (7.50)	
Clinical situation		
Severe clinical profile^a^	42 (35.00)	
Heart rate (beat/min)		89.00 [80.00–99.75]
> 45 and ≤ 120	112 (94.92)	
> 120	6 (5.08)	
Respiratory rate (/min)		21.50 [18.00–28.00]
> 30	20 (17.86)	
Systolic blood pressure (mmHg)		130.00 [120.0–143.5]
> 140	33 (28.45)	
Glasgow score		15.00 [15.00–15.00]
< 15	5 (4.67)	
Early Warning Score		6.00 [3.00–9.00]
≤ 4	44 (39.64)	
> 4 and ≤ 6	18 (16.22)	
> 6	49 (44.14)	
Biology		
Creatinine (µmol/L)		78.00 [61.50–94.50]
> 120	20 (16.81)	
CRP (mg/L)		92.00 [34.38–157.25]
< 40	33 (28.45)	
≥ 40 and < 150	52 (44.83)	
≥ 150	31 (26.72)	
Lymphocytes (G/L)		0.90 [0.60–1.20]
< 1.5	100 (84.03)	
Neutrophils (G/L)		5.10 [3.30–6.70]
< 2	4 (3.39)	
Bacterial co-infection	4 (3.33)	
Therapeutics		
Antivirals^b^	107 (89.17)	
Hydroxychloroquine	26 (24.30)	
Antibiotic therapy	115 (95.83)	
Corticoids	80 (66.67)	
Anticoagulants	111 (92.50)	
Evolution		
Transfer in ICU	35 (29.17)	
Oxygen therapy	52 (43.33)	
Pulmonary embolism	9 (7.50)	
Home visits	88 (73.33)	
Follow-up care and rehabilitation	31 (26.67)	
Re-hospitalisation	5 (4.17)	

^a^Includes severe initial pneumonia and acute respiratory distress syndrome (ARDS). Criteria for severe pneumonia were: fever and SpO_2_ < 90% or respiratory rate > 30/min or acute respiratory failure needing respiratory support (invasive or not) and/or admission in ICU and/or acute circulatory failure (sepsis or septic shock)

^b^Includes: Lopinavir/Ritonavir or Darunavir/Ritonavir or Remdesivir

The mean age of the sample was 63.50 years (IQR = [54.00–71.25]) and 54.17% were men. Most patients had symptoms of fever (76.67%), cough (73.33%) and dyspnoea (66.67%). A severe clinical profile, defined as ARDS or severe pneumonia (fever, SpO_2_ < 90% respiratory rate > 30/min, acute respiratory failure with respiratory support needed (invasive or non-invasive) or admission in ICU, associated acute circulatory failure (sepsis or septic shock)) was present for 35% of the patients.

Biological data showed a median lymphocyte count at 0.9 G/L (IQR [0.6–1.2]), a median neutrophil count at 5.1 G/L (IQR [3.3–6.7]) and a median CRP of 92.00 mg/L (IQR [34.38–157.25]).

Concerning evolution, 43.33% required oxygen therapy and 29.17% were transferred to the resuscitation unit. Antibiotic therapy was used for 95.83% of the patients, 92.5% of the patients received anti-coagulants and 66.6% received corticoid therapy.

At 6 months and 12 months, about 40% of the patients presented dyspnoea. The median mMRC score (at the two assessment) was 1 (IQR = [0–2]) and there was no significant difference between M6 and M12 (p value = 0.454).

The symptoms following the infection persisted at M6 and M12, with about 20% of the patients with cough and headache. Other symptoms were also observed (Table 2), anxiety, and problems with concentration and memory in particular.

**Table 2:** Description and comparison of states of health in assessments at M6 and M12

	M6 n = 120	M12 n = 120
Re-hospitalisation		
Yes (%)	4 (3.33)	10 (8.33)
Experienced dyspnoea		
Yes (%)	47 (39.87)	50 (41.67)
Experienced palpitations		
Yes (%)	19 (15.83)	19 (15.83)
Chest pains		
Yes (%)	19 (15.83)	18 (15)
Cough		
Yes (%)	24 (20.17)	25 (20.83)
Headaches		
Yes (%)	24 (20.17)	25 (20.83)
Arthromyalgia		
Yes (%)	49 (41.88)	56 (46.67)
Diarrhoea		
Yes (%)	19 (16.24)	20 (16.67)
Other symptoms*		
Yes (%)	42 (35.00)	54 (45.00)
Systolic blood pressure		
Median [Q1–Q3]	132.00 [120.00–145.0]	133.00 [120.00–149.00]
≤ 140 mmHg (%)	77 (65.81)	71 (61.21)
> 140 mmHg (%) (high blood pressure)	40 (34.19)	45 (38.79)
Heart rate (bpm)		
Median [Q1–Q3]	76.00 [67.00–88.00]	78.00 [66.00–85.00]
mMRC rating		
0 (%)	45 (37.50)	42 (35.59)
1 (%)	37 (30.83)	36 (30.51)
2 (%)	17 (14.17)	19 (16.10)
3 (%)	12 (10.00)	12 (10.17)
4 (%)	9 (7.50)	9 (7.63)
Anxiolytics		
Yes (%)	11 (9.32)	15 (12.50)
BMI		
Median [Q1–Q3]	28.00 [25.00–32.00]	28.00 [25.00–32.00]
≥ 40 kg/m^2^ (%)	9 (7.56)	7 (5.83)
< 40 kg/m^2^ (%)	110 (92.44)	113 (94.17)
SpO_2_		
Median [Q1–Q3]	97.00 [96.00–98.00]	96.00 [94.00–97.75]
< 94% (%)	2 (1.74)	6 (5.26)
> 94% (%)	113 (98.26)	108 (94.74)
Hypnotics		
Yes (%)	7 (5.93)	15 (12.5)
Antidepressants		
Yes (%)	11 (9.32)	17 (14.17)
Anticoagulants		
Yes (%)	19 (16.10)	18 (15.00)
Asthenia		
Yes (%)	69 (57.50)	69 (57.50)
FSS mean score		
> 4 (%)	39 (35.65)	51 (43.97)
< 4 (%)	72 (64.35)	65 (56.03)
Mean ± SD	3.42 ± 1.87	3.60 ± 1.97
SF12 score		
PCS12: mean ± SD	42.85 ± 10.03	42.18 ± 9.60
MCS12: mean ± SD	46.70 ± 7.57	47.13 ± 6.43

*BMI* Body Mass Index, *SpO*_*2*_ peripheral oxygen saturation

*Anxiety, problems of concentration, decreased visual acuity, panic attacks, rhinorrhea, fatigue, insomnia, cognitive decline, dyspnoea upon effort, hair loss vertigo, headaches, diarrhoea, constipation, dysgeusia

More than half of the patients at 6 and 12 months reported asthenia; the FSS fatigue scale was administered at both assessments, with a median of 3.11 (IQR [1.73–5.00]) at M6 and 3.22 (IQR [1.89–4.95]) at M12. Applying the threshold of the mean score calculated in the population, it was observed that 35% and 44% of the patients were experiencing fatigue at M6 and M12, respectively. The level of fatigue did not seem to differ between the two assessments (p value = 0.41), despite the tendency to increase.

Concerning quality of life, the SF12 was administered at M6 and M12, with a mean PCS12 score at 6 months of 42.85 (95% confidence interval (95% CI [41.05–44.65]) and a mean MCS12 score of 46.70 ((95% CI) [45.34–48.06]). There was no difference at M6 and M12 between the two SF12 scores: PCS12 and MCS12 with a p value of 0.417 and 0.501 respectively, although a slight improvement in the scores was observed at M12. Detailed results by SF12 components of patients assessed at M6 and at M12 were presented in Additional file 1: Table S1.

We found no differences between the results of scores taken by patients (SF12, FSS and mMRC) who presented a severe initial respiratory status versus those with a non-severe respiratory status. All p-values were greater than 0.05 (Table 3).

**Table 3:** SF12, FSS and mMRC scores comparison at 6 and 12 months follow up between severe and non-severe respiratory status at inclusion

	Severe^a^	Non-severe^b^	p value
6 months (N)	42	78	
SF12			
*PCS12* (*mean* ± *SD*)	42.7 ± 9.7	42.9 ± 10.2	0.87
*MCS12* (*mean* ± *SD*)	46.6 ± 7.8	46.7 ± 7.5	0.87
FSS			
(*Mean* ± *SD*)	29.8 ± 17.0	31.4 ± 16.9	0.64
mMRC			
(*Median*, *Q*1–*Q*3)	1, 0–1.8	1, 0–2	0.67
12 months (N)	42	78	
SF12			
*PCS12* (*mean* ± *SD*)	42.2 ± 9.1	42.2 ± 9.8	0.92
*MCS12* (*mean* ± *SD*)	48.0 ± 6.4	46.7 ± 6.4	0.16
FSS			
(*Mean* ± *SD*)	29.5 ± 17.1	34.0 ± 17.9	0.22
mMRC			
(*Median*, *Q*1–*Q*3)	1, 0–1	1, 0–2	0.45

^a^Includes severe initial pneumonia and acute respiratory distress syndrome (ARDS). Criteria for severe pneumonia were: fever and SpO_2_ < 90% or respiratory rate > 30/min or acute respiratory failure needing respiratory support (invasive or not) and/or admission in ICU and/or acute circulatory failure (sepsis or septic shock)

^b^Includes respiratory symptoms without identified pneumonia

**Fig. 2:**
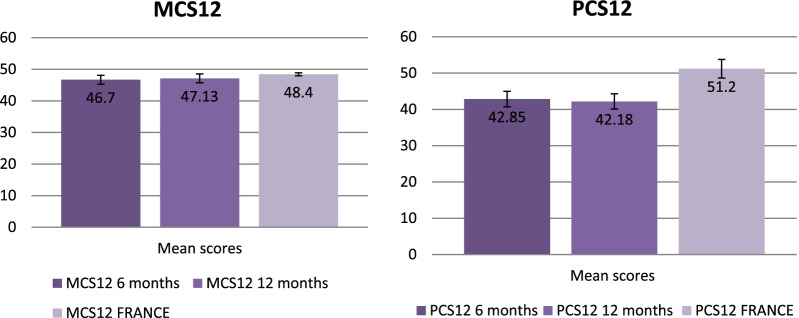
Comparison of PCS12 and MCS12 scores from the SF12, for patients at M6 and M12 versus the French population (means with CI 95%)

Finally, we compared the mean SF12 scores with those in the general population in France (Fig. 2) [19]. The general population was composed of 48% of males and the mean age (SD) was 44.6 (18.1) years [20]. We observed lower scores among Covid-19 patients than in the general population, meaning that quality-of-life in both the physical and the psychological dimensions was poorer (p value < 0.001 for the PCS12 at M6 and M12 versus the norm, and p value 0.025 and 0.037 respectively for the MCS12 assessed at M6 and M12, versus the norm) [19].

## Discussion

To our knowledge, this study was one of the first to describe the health state of Covid-19 patients at 6 and 12 months including assessments of quality-of-life and persistent fatigue. It highlighted persistent dyspnoea, with an mMRC score above 1 for more than half of the patients. The FSS score also evidenced persistent fatigue for 35% of the patients at M6 and 43% at M12. The two SF12 scores, PCS12 and MCS12, evidenced physical and mental quality-of-life scores below the mean for the French population. For all these scores, no difference was observed between the two time-points. This could therefore suggest the long-term persistence of certain symptoms, with general fatigue and poorer quality-of-life than the norm, both physical and mental.

Certain studies have described and compared patients’ state of health at 6 months after hospital discharge [4] and others at 6 and 12 months [8, 21, 22]; all these studies have reported the presence of fatigue and a change in quality-of-life among patients after hospital discharge. Only one study used a quality-of-life assessment (HRQoL) [8], but none used the FSS score or the SF12 score. However, the results from all these studies and from ours concur on the fact that the main symptoms present after infection by SARS-CoV2 were fatigue, dyspnoea, anxiety and repercussions on cognitive functions [8, 21, 22]. In our study, we focused particularly on the patients’ clinical state, using the SF12 score to determine global quality-of-life. Our results mainly suggest stability in their clinical state between the two time-points (M6 and M12), thus pointing to a period of latency between the end of the infection and a return to what is considered a normal life. Our study was conducted early on and did not have the privilege nor the objective to determine the effect of vaccination on quality of life since the patients were not all vaccinated at assessment. Our population was older and included more men than the population used for comparison.

This study thus showed a non-negligible decrease in the patients’ physical and mental quality-of-life at 6 months and 1 year following infection by SARS-CoV2, compared to the French general population. It showed persistence, 1 year after the initial infection, of fatigue and dyspnoea, alongside the appearance of psycho-neurological symptoms.

These observations raise several issues, in particular that concerning the evolution of the disease and the “post-COVID” period. They are also in favour of the “Long Covid” hypothesis. This condition was defined by the French health authorities [23] as confirmed SARS-CoV2 infection with the initial symptoms persisting beyond 4 weeks after the onset of the acute phase, and not explained by another diagnosis.

While it is easy to incorporate fatigue into the notion of “Long Covid”, the fatigue and the decrease on quality-of-life observed could also be explained by a psychiatric dimension [24]. Indeed, certain studies have found possible neuro-psychiatric effects, or even an aggravation of certain psychiatric pathologies that were already present, following infection with SARS-CoV2, as with other already known viral infections [24, 25].

This overlap between infection by SARS-CoV2 and a probable cerebral inflammation could explain in part feelings of ill being or chronic fatigue. Perceived quality of life could thus be affected despite a satisfactory physical state in clinical consultation.

These hypotheses should be explored and assessed for COVID-19, but they can already explain the decline in quality-of-life, especially for the psychological sphere, observed among patients, sometimes discordant with the physical state [24].

We were able to maintain the same number of patients between the two time-points. All the scales and tools used to assess fatigue and quality-of-life were standardised and validated. The researchers were experienced specialist doctors, which enabled reliable data collection. Furthermore, the patients were seen at 6 and 12 months by the same researchers, enabling comparable data collection between the two assessments.

## Conclusions

This study was one of the first descriptions of the state of health of COVID-19 patients with certain persistent symptoms: fatigue and deterioration in physical and mental quality-of-life. This information could guide care providers in the follow-up care provided to patients and in rehabilitation, and if needed, in neuro-psychiatric care.

## Supplementary Information

**Additional file 1: Table S1.** Detailed results by SF12 components of patients assessed at M6 (n = 120) and at M12 (n = 116).

## Data Availability

The datasets generated and/or analysed during the current study are not publicly available due to European General Data Protection Regulation and medical secrecy but are available from the corresponding author on reasonable request.

## Abbreviations

COVID-19: CoronaVirus Disease 2019
SARS CoV2: Severe Acute Respiratory Syndrome-Coronavirus 2
ARDS: Acute respiratory distress syndrome
WHO: World Health Organisation
mMRC: Modified Medical Research Council score
FSS: Fatigue Severity Scale
SF12: Short Form 12
PCS12: Physical Component Summary score
MCS12: Mental Component Summary score
IQR: InterQuartile Range
