# Supplementary material for: Fatigue and quality-of-life in the year following SARS-Cov2 infection
Source: BMC Infect Dis. 2022 Jun 13;22:541. doi: 10.1186/s12879-022-07517-w (PMC9189445; doi:10.1186/s12879-022-07517-w)
Supplement: Supplementary file 1 — Additional file 1: Table S1. Detailed results by SF12 components of patients assessed at M6 (n = 120) and at M12 (n = 116). [file 12879_2022_7517_MOESM1_ESM.docx]

**Additional table S1:** Detailed results by SF12 components of patients assessed at M6 (n=120) and at M12 (n=116)

**Components Number (%)**

**M6 M12**

**PCS12**

**General Health (GH)**

Excellent 2 (1.6) 4 (3.4)

Very good 18 (15.0) 14 (12.1)

Good 65 (54.17) 65 (56.0)

Fair 26 (21.7) 27 (23.3)

Poor 9 (7.5) 6 (5.2)

**Physical Functioning (PF)**

**Moderate activities (such as moving a table, pushing a vacuum cleaning, bowling…)**

Yes, limited a lot 18 (15.0) 16 (13.8)

Yes, limited a little 44 (36.7) 44 (37.9)

No, not limited at all 58 (48.3) 56 (48.3)

**Climb several flights of stairs:**

Yes, limited a lot 35 (29.2) 33 (28.5)

Yes, limited a little 54 (45.0) 52 (44.8)

No, not limited at all 28 (23.3) 31 (26.7)

ND 3 (2.5) 0 (0.0)

**Role Physical (RP)**

**Accomplished less than you would like**

All of the time 4 (3.3) 5 (4.3)

Most of the time 17 (14.2) 11 (9.5)

Some of the time 16 (13.3) 23 (19.8)

A little of the time 50 (41.7) 41 (35.4)

None of the time 33 (27.5) 36 (31.0)

**Were limited in the kind of work or other activities**

All of the time 2 (1.6) 4 (3.5)

Most of the time 20 (16.7) 10 (8.6)

Some of the time 15 (12.5) 21 (18.1)

A little of the time 47 (39.2) 51 (43.9)

None of the time 33 (27.5) 30 (25.9)

ND 3 (2.5) 0 (0.0)

**Bodily Pain (BP)**

**During the past 4 weeks, how much did pain interfere with your normal work?**

Not at all 40 (33.3) 32 (27.6)

A little bit 32 (26.7) 25 (21.5)

Moderately 31 (25.8) 30 (25.9)

Quite a bit 10 (8.4) 23 (19.8)

Extremely 7 (5.8) 6 (5.2)

**MCS12**

**Role Emotional (RE)**

**Accomplished less than you would like**

All of the time 4 (3.3) 3 (2.6)

Most of the time 13 (10.8) 7 (6.0)

Some of the time 24 (20.0) 19 (16.4)

A little of the time 32 (26.7) 41 (35.3)

None of the time 47 (39.2) 46 (39.7)

**Did work or activities less carefully than usual**

All of the time 4 (3.3) 0 (0.0)

Most of the time 16 (13.3) 14 (12.1)

Some of the time 15 (12.5) 17 (14.7)

A little of the time 42 (35.0) 41 (35.3)

None of the time 43 (35.9) 44 (37.9)

**Mental Health (MH)**

**Have you felt calm and peaceful?**

All of the time 10 (8.3) 9 (7.7)

Most of the time 39 (32.5) 38 (32.8)

Some of the time 23 (19.2) 26 (22.4)

A little of the time 40 (33.3) 27 (23.3)

None of the time 8 (6.7) 15 (12.9)

ND 0 (0.0) 1 (0.9)

**Have you felt downhearted and depressed?**

All of the time 3 (2.5) 2 (1.7)

Most of the time 8 (6.7) 11 (9.5)

Some of the time 16 (13.3) 11 (9.5)

A little of the time 43 (35.8) 51 (44.0)

None of the time 50 (41.7) 36 (31.0)

ND 0 (0.0) 1 (0.9)

**Vitality (VT)**

**Did you have a lot of energy?**

All of the time 2 (1.7) 3 (2.6)

Most of the time 14 (11.7) 13 (11.2)

Some of the time 15 (12.5) 11 (12.1)

A little of the time 52 (43.3) 57 (49.1)

None of the time 37 (30.8) 28 (24.1)

ND 0 (0.0) 1 (0.9)

**Social Functioning (SF)**

**During the past 4 weeks, how much of the time has your physical health or emotional problems interfered with your social activities (like visiting friends, relatives, etc.)?**

All of the time 3 (2.5) 3 (2.6)

Most of the time 6 (5.0) 7 (6.1)

Some of the time 15 (12.5) 13 (11.3)

A little of the time 38 (31.7) 38 (33.0)

None of the time 57 (47.5) 53 (46.1)

ND 1 (0.8) 1 (0.9)
